# Editorial: Gut microbiota's role in high-altitude animal adaptation

**DOI:** 10.3389/fmicb.2025.1613028

**Published:** 2025-05-29

**Authors:** Lin Zhang, Wei Zhu, Kashif ur Rehman, Huan Li

**Affiliations:** ^1^School of Basic Medical Sciences, Hubei University of Chinese Medicine, Wuhan, China; ^2^Chengdu Institute of Biology, Chinese Academy of Sciences, Chengdu, China; ^3^Department of Microbiology, Faculty of Veterinary and Animal Sciences, The Islamia University of Bahawalpur, Bahawalpur, Pakistan; ^4^German Institute of Food Technologies (DIL e.V.), Quakenbrück, Germany; ^5^School of Public Health, Lanzhou University, Lanzhou, China

**Keywords:** gut microbiota, adaptation, high-altitude environment, host-microbiota interaction, physiological function

The adaptation of animals to high-altitude habitats has interested scientists for an extended period, owing to the challenging conditions like hypoxia, cold temperatures, elevated sun radiation, and limited food resources (Chang et al., 2025). Historically, research concentrated on physiological and genetic adaptations (Storz, 2021). However, an expanding body of information also highlights another equally strong direction of adaptation: the gut microbiome. The gut microbiota (microbiome) is known as the second genome (Weinstock, 2012), this sophisticated and dynamic community of microbes is essential for host digestion, metabolism, immunity, and general survival under adverse settings (Berg, 2014; Hale et al., 2018; Turner, 2018; Chen et al., 2024; Wang et al., 2024), Animals and their gut microbiota have co-evolved (Montoya-Ciriaco et al., 2020), forming mutualistic relationships that are crucial for various life activities, including digestion, metabolism, and immunity (Kohl et al., 2017; Zhang L. et al., 2021; Zhang et al., 2022; Bahadur et al., 2024). This editorial shows innovative findings across several species, including macaques, moles, sheep, and cranes, eliminating the essential function of gut microbiota in facilitating survival and health at high elevations.

High-altitude environments not only affect animals but also their gut microbiota. Comparative microbiota analyses of Yorkshire and Tibetan pigs (Liu et al.), it indicated that Tibetan pigs exhibit higher abundances of phyla *Actinobacteria, Fibrobacteres*, and genus *Rhodococcusi*, while showing lower abundances of phyla *Bacteroidota* and *Spirochaetota*. The enrichment of *Rhodococcus* may enhance the metabolic detoxification capabilities and produce short-chain fatty acid (SCFA). Compared to Hu sheep, Tibetan sheep exhibit significantly greater rumen epithelial nipple height and significantly smaller nipple width (Chen et al.). Tibetan sheep show a notably higher abundance of phyla *Bacteroidetes*, which facilitates efficient nutrient utilization, while the abundances of *Firmicutes* and *Patescibacteria* are significantly lower than those in Hu sheep. The functional relationship between the host's gastrointestinal structure and microbial inhabitants constitutes a complex adaptive strategy. Compared to *Lasiopodomys brandtii, Meriones unguiculatus, Ochotona curzoniae*, and *Spermophilus dauricus*, the gut microbiota in *Myospalax baileyi* shows a significant enrichment of phyla *Firmicutes* and *Bacteroidetes*, including *Cellulosilyticum lentocellum*, which aids in dietary fiber breakdown. Additionally, the gut microbiota of *M. baileyi* is enriched with SCFA producing genera such as *Lachnospiraceae, Pseudoflavonifractor, Eubacterium, Flavonifractor, Clostridium*, and other, which may mitigate inflammatory responses induced by low pressure, hypoxia, and high concentrations of CO_2_, facilitating high-altitude adaptation (Hu et al.). The gut microbiota diversity exhibits significant complexity between low-altitude and high-altitude individuals. A comparative analyses of wild macaques, humans, and dogs living at different altitudes, the high-altitude population exhibited greatest degree of microbial convergence, principally due to dietary overlap and environmental exposure. Microbial diversity was also highest in wild macaques with more complex and varied diets. High-altitude environments promoted microbial sharing among species, which is explained by environmental factors and human-mediated contact facilitating interspecific cross-species microbiota transmission. This may be a fundamental strategy to improve the adaptive potential in numerous host species co-occurring within comparable ecological niches (Zhao J. et al.). A comparison of the oral saliva microbiomes in Han Chinese populations indicated that altitude is a significant factor affecting microbial compositions and metabolic profiles of the human oral cavity. High-altitude participants demonstrated more notable abundance in genera such as *Selenomonas* and *Prevotella*, whereas low-altitude phenotypes represented higher abundances of *Haemophilus* and *Neisseria*. Several observed microbial shifts were associated with distinct alterations in genes and pathways of energy, amino acid, and carbohydrate metabolism and nearly 1,000 unique metabolites across different compound classes. Correlation analyses also identified potential interactions between specific microbes and metabolic products, thus illustrating the pervasive effects of altitude on human microbiome-host interaction involving sites other than the gut (Li et al.). However, compared to low-altitude populations, the gut microbiota of *Bombus pyrosoma* in high-altitude regions exhibits reduced alpha diversity (with lower Chao1 and Faith-pd index) and a more uniform community composition, likely due to limited diversity in extreme environments (Zhang Z. et al.). Investigating soil microbiota in marmot-habitat represents an excellent example for a better understanding of the role and complexity of microbial communities affecting host-pathogen dynamics. Different soil properties and, thereby, different enrichment of microbial taxa in the soils are characteristic of Marmota habitats, which may also positively influence survival characteristics of Yersinia pestis against other hosts such as pikas. This reflects a more basic microbiome-host-disease relationship where the gut flora may be important as mediating adaptation and susceptibility to disease (Zhao W. et al.).

Gut microbiota exhibit pronounced seasonal patterns across diverse animal species, reflecting adaptations to environmental and dietary changes. The seasonal variations in the gut microbiota of migratory black-necked cranes (*Grus nigricollis*) indicate their adaptation to realized ness across a year. Summer scores the highest microbial diversity and functional richness via *Faprotax*, with an evenly balanced community structure, whereas *Lactobacillacea* is predominant in other seasons through analysis of fecal samples. Although the community had higher diversity in summer, the microbial network was structurally simpler than in winter, which might reflect seasonality constraints on dispersal affecting the assembly of microbial communities. Our results exemplify how changes in the environment (and associated diet) can influence migratory bird gut microbial assemblage, underline the adaptability of avian microbiomes, and overall highlight its role within an eco-evolutionary framework (Zhang Y. et al.). Cold-tolerant min pigs rely on their microbiome to drive the upregulation of vitamin B1 biosynthesis for this characteristic. The winter-fortified Bacteroidetes-dominant gut microbiome exhibited six significantly enriched thiamine synthetase genes. These shrub-associated microbial genes belong to functional categories under purifying selection, suggesting a direct impact on host energy metabolism in the cold. This is also consistent with the presence of these genes only in Min pigs (but not Duroc pigs), indicating that host genotype-microbial functions influence each other and further demonstrating the importance of a specific relationship between animal hosts and *ex vivo* microbiota composition during climate selection (Chang et al.).

Previous studies have demonstrated a strong positive correlation between the elevation and the proportion of strictly anaerobic bacteria in the gut microbiota of wild animals (Suzuki et al., 2018; Li et al., 2019). However, in *Nannospalax xanthodont* across six locations spanning three altitudinal groups (Solak et al.), there is no significant difference in strictly anaerobic bacteria abundance among altitudinal groups, despite facultatively anaerobic bacteria being more prevalent at higher altitudes. In *Cricetulus longicaudatus*, individuals from high-altitude regions individuals significantly lower beta diversity of gut microbiota compared to those from low-altitude area, altitude was strongly associated with shifts in microbial composition, showing a negative connection with norank_*Muribaculaceae* and a positive correlation with *Providencia* (Ren et al.). Similarly, captive sheep and deer show shifts in dominant phyla, as *Firmicutes, Bacteroidetes*, and *Proteobacteria*, though with taxon-specific patterns. A comparative analysis of fecal microbiota from six captive deer, including *Cervus canadensis, C. elaphus, C. nippon, Dama dama, Elaphurus davidianus*, and *Rangifer tarandus, Firmicutes* and *Bacteroidetes* were consistently predominant, but *E. davidianus*, fed a starch-rich diet, displayed significantly higher *Proteobacteria* and lower *Bacteroidetes* than other species (Zhao C. et al.), results reveal essential discoveries regarding microbiota-driven physiology and establish a framework for new conservation and management guidelines to support captive deer populations. This aligns with findings in Tibetan Awang sheep (*Ovis aries*), where the entire captivity group (fed concentrates) had elevated phyla *Proteobacteria*, notably *Acinetobacter*, a potential pathogen, compared to semi-captivity or grazing groups, while *Bacteroides* abundance correlated positively with altitude, suggesting its adaptive role in energy harvest at high-altitude (Wang et al.).

The gut is the primary site for absorbing and reformulating nutrients from food (Moran et al., 2019; Martínez-Mota et al., 2020; Montoya-Ciriaco et al., 2020). In *Eothenomys miletus*, the dominant fecal microbial phyla are *Firmicutes, Bacteroidetes*, and *Spirochaetes* (Zhang W. et al.; Jia et al.; Zhang T. et al.). Seasonal variation in food resources significantly influence physiological traits such as body mass, brown (BAT) and white (WAT) adipose tissue mass and resting metabolic rate (RMR) changed when *E. miletus* faces food shortage. Interestingly, while food restriction dose not significantly influence gut microbiota's alpha or beta diversity (Zhang W. et al.), it induces specific microbial adaptations. To optimize nutrient utilization under food shortage, the gut microbiota of *E. miletus* enhances the genera of *Bacterroides, Ruminococcus, Turicibacter*, and *Treponema*. *Ruminococcus* abundance typically increases with high fiber diets, as this genus plays a key role in fiber degradation. *Turicibacter* modulates bile acids and lipids, contributing to reduced adipose tissue mass and serum cholesterol level (Lynch et al., 2023). *Treponema* is associated with cellulose degradation (Baniel et al., 2021), which is particularly relevant for *E. miletus*, as their diet primarily consists of high-fiber plants. In low-altitude population, high fiber diets enrich *Alistipes, Sporobacter*, and *Rikenellaceae*, which are involved in SCFA metabolism and aromatic compound degradation. In contrast, high-altitude population show enrichment of *Anoxybacillus* and *Methylobacteriaceae*, with *Anoxybacillus* promoting the proliferation of probiotics like *Lactobacillus* (Liu et al., 2021). These microbial shifts enhance fiber digestion and energy provision. However, the consumption of high-fat food significantly increased body mass, WAT mass, and serum leptin levels in *E. miletus*, thought it did not alter food intake (Jia et al.). High fat diets also reduced the RMR and shifted energy metabolism toward lipid utilization (Gong et al., 2021). At the microbial level, high fat food changed the gut microbiota composition, particularly affecting alpha diversity but not beta diversity in *E. miletus* (Jia et al.).

Analysis of the gut microbiota in high-altitude populations reveals significant composition and diversity shifts under hypoxic, including an increase in *Blautia* A, which supports microbial stability and host adaptation to low-oxygen conditions (Cheng et al., 2022). Notably, *Lactobacillus johnsonii* HL79 treatment improved cognitive function, as evidenced by increased exploration ratios in the NOR test compared to controls. Microbiota analysis identified six key phyla *Firmicutes, Bacteroidetes, Verrucomicrobia, Actinobacteria, Proteobacteria*, and *Epsilonbacteraeota*, with *Firmicutes* significantly higher in control group than in high-altitude group exposed mice. Conversely, *Bacteroidetes* exhibited pronounced abundance changes in the HL79-treated high-altitude group, indicating responsiveness to probiotic intervention (Zhao Z. et al.).

The research in this Research Topic stresses that gut microbiota is more than an indifferent spectator but an active and changeable player to high-altitude adaptation. These microbial consortia help coordinate elaborate physiological functions that promote host survival in some of the severest environments on Earth, whether through increasing nutrient uptake or modulating immune responses, promoting thermogenesis, maintaining cognitive function, etc. These converging lines of evidence suggest an evolutionary problem in which adaptational processes within the microbiome are a central, though likely not sole or primary driver during acclimatization to high-altitude (Luo et al., 2024). The future of this field lies in the experimental confirmation of host-microbiota interactions and translating these findings into applied solutions for livestock agriculture, wildlife conservation, as well even interventions that support humans adapting to extreme environments.
